# Is premorbid scapulohumeral rhythm restored with anatomic or reverse shoulder arthroplasty for cuff-intact osteoarthritis? An in-vivo dynamic radiography study

**DOI:** 10.1016/j.jseint.2025.06.008

**Published:** 2025-07-04

**Authors:** Zaamin B. Hussain, Sameer R. Khawaja, Musab Gulzar, Jaden C. Hardrick, Krishna N. Chopra, Anna Gorsky, Victoria A. Conn, Michael B. Gottschalk, Eric R. Wagner

**Affiliations:** a Department of Orthopaedic Surgery, Emory University School of Medicine, Atlanta, GA, USA; b Department of Orthopaedic Surgery, Baylor College of Medicine, Houston, TX, USA

**Keywords:** Anatomic total shoulder arthroplasty, Reverse shoulder arthroplasty, Primary glenohumeral osteoarthritis, Dynamic radiography, Scapulohumeral rhythm, Shoulder biomechanics

## Abstract

**Background:**

Anatomic total shoulder arthroplasty (aTSA) and reverse total shoulder arthroplasty (rTSA) are both treatment options for advanced glenohumeral osteoarthritis with an intact rotator cuff; however, decision making is controversial, especially among younger active patients. Restoring native shoulder kinematics may be an important consideration for implant longevity and ultimate shoulder function, but *in-vivo* assessment and comparisons have been historically difficult. The purpose of this study was to compare scapulohumeral rhythm (SHR) between aTSA and rTSA when performed for patients with cuff-intact osteoarthritis and compare these with preoperative values and normal controls.

**Methods:**

A retrospective analysis was performed on 71 shoulders that underwent arthroplasty for cuff-intact osteoarthritis, aTSA (n = 28) and rTSA (n = 43), who had dynamic digital radiography performed more than 6 months postoperatively and compared these to 32 normal controls. SHR was calculated by dividing the change in glenohumeral abduction (ΔH) by the change in scapular upward elevation (ΔS) using the formula SHR = ΔH/ΔS, across the total range of abduction below 120° and between the 0°-30°, 30°-60°, 60°-90°, and 90°-120° abduction intervals. A paired subgroup analysis was performed on 14 aTSA and 14 rTSA shoulders with both pre- and postoperative dynamic digital radiography. Descriptive statistics were used to summarize data and differences between groups were analyzed using unpaired Student's *t*-tests for continuous variables, and a paired *t*-test for subgroup analyses, as well as a Bonferroni correction for multiple statistical tests. Interclass correlation of measurements was used to calculate the inter-rater reliability between the two measurers. All analyses were carried out using R v. 3.6.1. (R Foundation for Statistical Computing, Vienna, Austria). A *P* value of less than .05 was considered statistically significant.

**Results:**

The aTSA cohort had a similar median rest–120° SHR of 2.00 compared to 1.95 for the rTSA cohort (*P* = .948), but both were lower than normal controls with a SHR of 2.38 (*P* < .001). Preoperative vs. postoperative analyses of the aTSA and rTSA cohorts show significant improvements in preoperative to postoperative median rest–120° SHR from 1.36 to 2.10 (*P* = .0002) and 1.34 to 2.04 (*P* = .002), respectively. The inter-rater reliability was 0.99.

**Conclusion:**

Patients who underwent aTSA and rTSA for rotator cuff–intact glenohumeral osteoarthritis are associated with lower SHRs than normal asymptomatic patients; however, SHRs significantly improved from preoperative levels. There was no difference between postoperative SHRs for rTSA and aTSA. aTSA and rTSA both partially restore coordination between the glenohumeral and scapulothoracic joints, although not to the extent of normal healthy shoulders.

The incidence of shoulder arthroplasty has been rising exponentially in the last decade.^43^ Both anatomic total shoulder arthroplasty (aTSA) and reverse total shoulder arthroplasty (rTSA) prostheses have demonstrated clinical success and comparable outcomes for treating glenohumeral osteoarthritis with an intact rotator cuff.^8^^,^^12^^,^^33^^,^^34^^,^^42^ Maximizing impingement-free range of motion is considered critical to optimize functional outcomes in aTSA and rTSA. Recent advances have begun to uncover the important role of restoring more normal scapula biomechanics for improved shoulder coordination after arthroplasty. In addition, reducing glenohumeral joint forces and bonet–implant interface strain may be protective for implant longevity,^2^^,^^6^ and restoring near-normal scapula biomechanics, soft tissue tension, and musclet–tendon lengths after arthroplasty may improve survivorship.^27^

The contribution of scapular motion is poorly understood in the setting of glenohumeral arthritis and after intervention with aTSA and rTSA. This could explain variability in outcomes between patients, as well as being potentially modifiable with rehabilitation.^30^ Understanding the differences in the scapula biomechanical alterations between rTSA and aTSA may also help inform a clinical algorithm in patients with cuff-intact glenohumeral arthritis, especially in younger more active patients. However, cadaveric models are unable to reproduce *in vivo* kinematic tension and load sharing, and computational models can be limited by assumptions and oversimplifications.^32^ *In-vivo* biomechanical comparison may help us understand how aTSA and rTSA influence motion and function at the glenohumeral and scapulothoracic articulations. Dynamic digital radiography (DDR) overcomes this limitation by using low-dose pulsed radiographs during patient motion, to produce dynamic radiographic sequences, and has been used in the shoulder to measure scapulohumeral rhythm (SHR).^14^^,^^15^^,^^19^^,^^20^^,^^39^^,^^45^ SHR is a ratio between glenohumeral and scapulothoracic motion, with higher values indicating more glenohumeral motion and lower values indicating more scapular motion, with normative values reported between 2.2 and 2.7.^3^^,^^10^^,^^28^^,^^45^ SHR is known to be low in patients after aTSA and rTSA,^37^ but it is not clear if this is a result of prior glenohumeral pathology or the intervention itself, as there is sparse literature on pre- to postoperative changes in SHR after shoulder arthroplasty.^11^ Advancing knowledge in this area may also provide useful information to aid arthroplasty planning, including target goals for glenohumeral motion, as well as informing postoperative rehabilitation regimens.

The purpose of this study was to measure SHR in patients who underwent aTSA and rTSA for cuff-intact glenohumeral arthritis and compare this to preoperative values in a paired subgroup analysis. We hypothesized that SHR after aTSA would be higher compared to rTSA, by having a larger glenohumeral contribution, which is more typical of native shoulders.

## Methods

### Patient inclusion

After institutional review board approval, a retrospective analysis with prospective follow-up was performed on patients with a primary diagnosis of advanced glenohumeral osteoarthritis with an intact rotator cuff, confirmed based on intraoperative findings, who underwent either primary aTSA or rTSA by a fellowship-trained shoulder surgeon between the years of 2019 and 2023. DDR was performed on patients following either aTSA or rTSA as per standard of care and part of the clinical workflow at this institution. Patients were included in this study if they had a postoperative DDR performed at greater than 6 months postoperatively. We used this time cut-off as a threshold, as shoulder kinematics in aTSA^13^ and rTSA^29^ are thought to not change significantly after 6 months postoperatively. Patients were excluded if there was history of prior surgery on the operative shoulder. Patients were compared to a group of asymptomatic normal native shoulder controls.

### Surgical technique

For aTSA, a lesser tuberosity osteotomy was performed and repaired using transosseous repair techniques. The humeral head cut was performed at the native retroversion. All cases used the Stryker Tornier Anatomic Perform (Stryker, Kalamazoo, MI, USA) Cortiloc glenoid and Simpliciti (Stryker, Kalamazoo, MI, USA) humeral stemless component. When necessary, the glenoid was augmented to within 5 degrees of neutral version with off-the-shelf augments. Postoperative rehabilitation followed standard aTSA protocol, with 4 weeks in a sling immobilization followed by physical therapy.

For rTSA, a subscapularis peel was performed and repaired using transosseous repair techniques. All cases used the Stryker Tornier Perform baseplate, glenosphere, and humerus. Using the center of the glenoid as reference, the guide pin and ultimately baseplate was then placed into the glenoid vault based on preoperative templating, aiming to be within 5 degrees of neutral version and inclination. The appropriately sized glenosphere was then placed, depending on the tension between the baseplate and humerus and size of the patient. With the glenoid in place, a stemmed inlay or onlay humeral component was then placed in standard fashion. Postoperative rehabilitation followed standard rTSA protocol, with 4 weeks in a sling immobilization followed by physical therapy.

### Digital dynamic radiography

**Figure 1:**
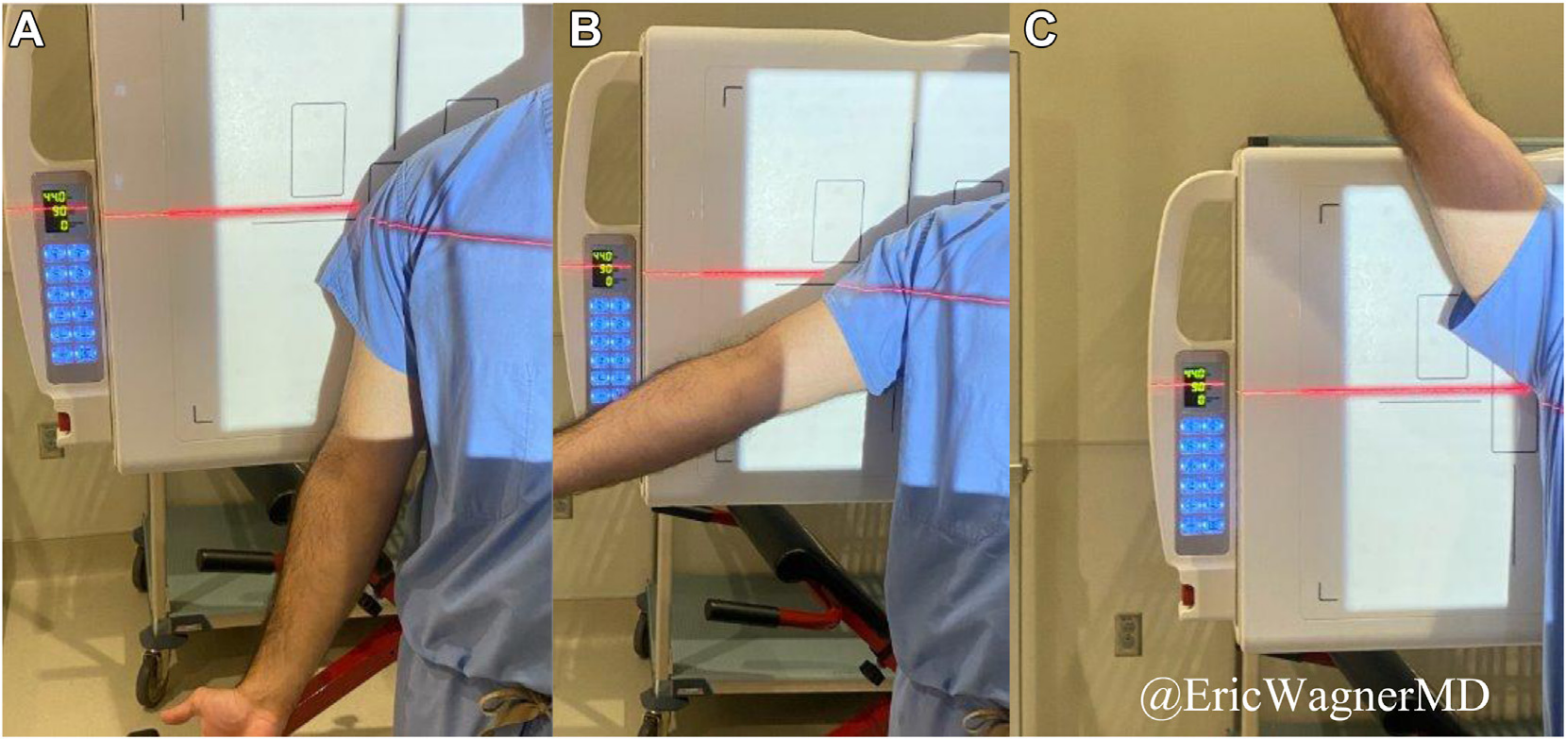
Procedure for capturing DDR images (**A-C**). Patients are positioned in a Grashey view with the posterior shoulder and scapula in contact with the back of the DDR machine. Each patient was instructed to stand upright, stationary, and avoid axial rotation, while following a video providing coordinated instructions on how to perform the motion, standardizing for forearm supination. Patients begin with their arm at rest (**A**) and gradually abduct their arm to maximum abduction (**C**). *DDR*, dynamic digital radiography.

**Figure 2:**
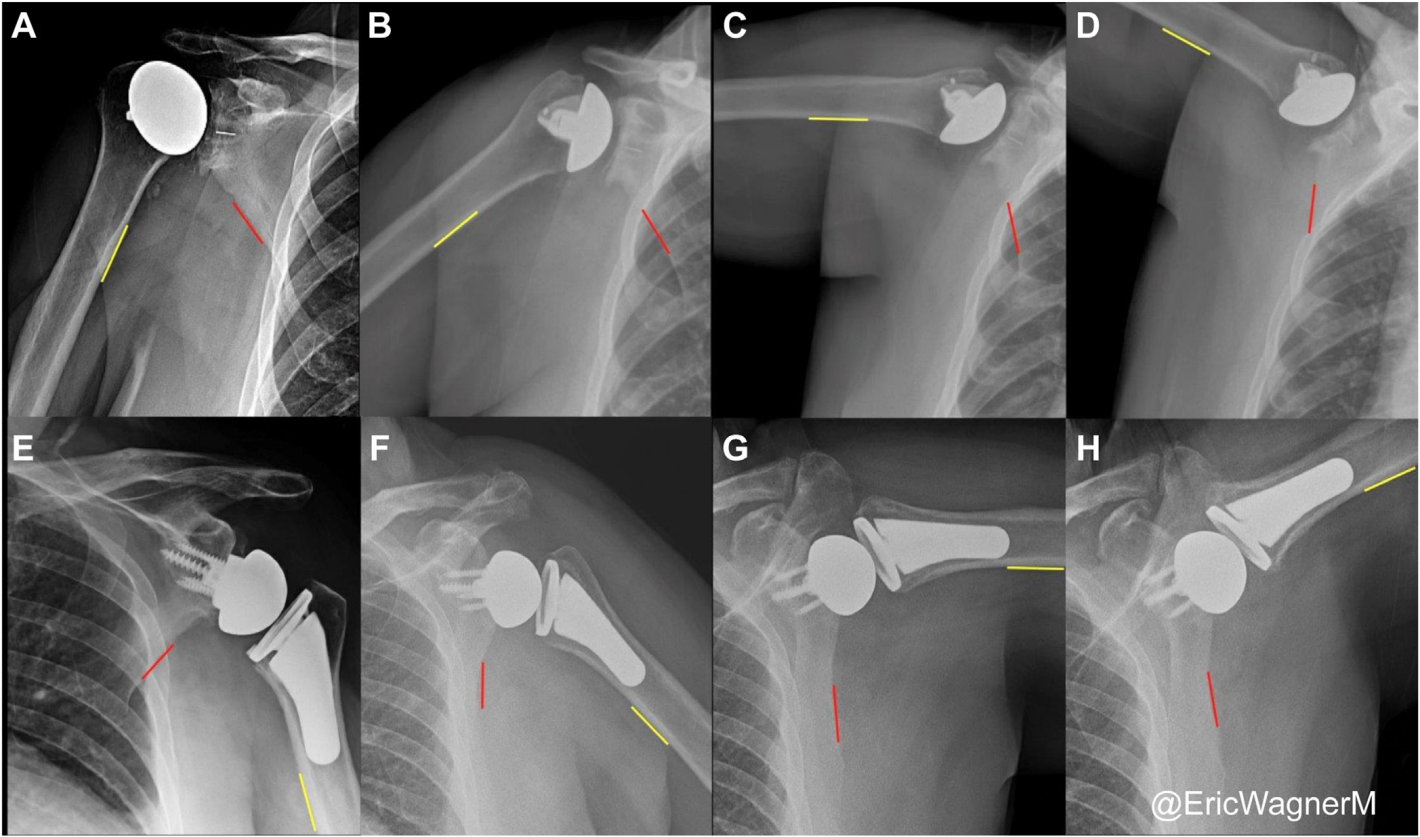
Radiographs showing glenohumeral and scapulothoracic abduction in a patient with aTSA (*top row*) and rTSA (*bottom row*) at rest (**A** and **E**), 45° abduction (**B** and **F**), 90° abduction (**C** and **G**), and 120° abduction (**D** and **H**). *aTSA*, anatomic total shoulder arthroplasty; *rTSA*, reverse total shoulder arthroplasty. *Red line* represents lateral border of the scapula, *yellow line* represents medial border of humerus.

All shoulders in the study were imaged and analyzed using DDR: Advanced U-Arm System (Konica Minolta, Wayne, NJ, USA) as per standard of care (Fig. 1). DDR obtains a series of pulsed radiographs ranging from 6 to 15 Hz for up to 20 seconds during shoulder motion (Fig. 2, Video 1, Video 2). An average DDR shoulder exam includes an entrance surface dose of ration exposure of 1.33 mGy, comparable (∼1.3x) to a static 2-view shoulder plain radiograph.^31^ The motion of interest was maximal arm abduction from rest in the plane of the body with the patient standing upright. Grashey views were acquired for a view perpendicular to the plane of glenohumeral motion. Dynamic images were taken under the supervision of one of three licensed radiology technicians who were trained using a standardized protocol. The radiology technicians coached patients on timing and cadence during required shoulder motions. Each patient is positioned with their posterior shoulder and scapula contacting the back board of the DDR machine. Patients are instructed to stand upright, stationary, and avoid axial rotation, and on how to perform the motion, including standardizing for forearm supination. Patients begin with their arm at rest and gradually abduct their arm to maximum abduction. The DDR images were analyzed by a fellowship-trained shoulder surgeon, and the images were read by fellowship-trained musculoskeletal imaging radiologists with greater than 5 years of independent reading experience.

### DDR measurements

**Figure 3:**
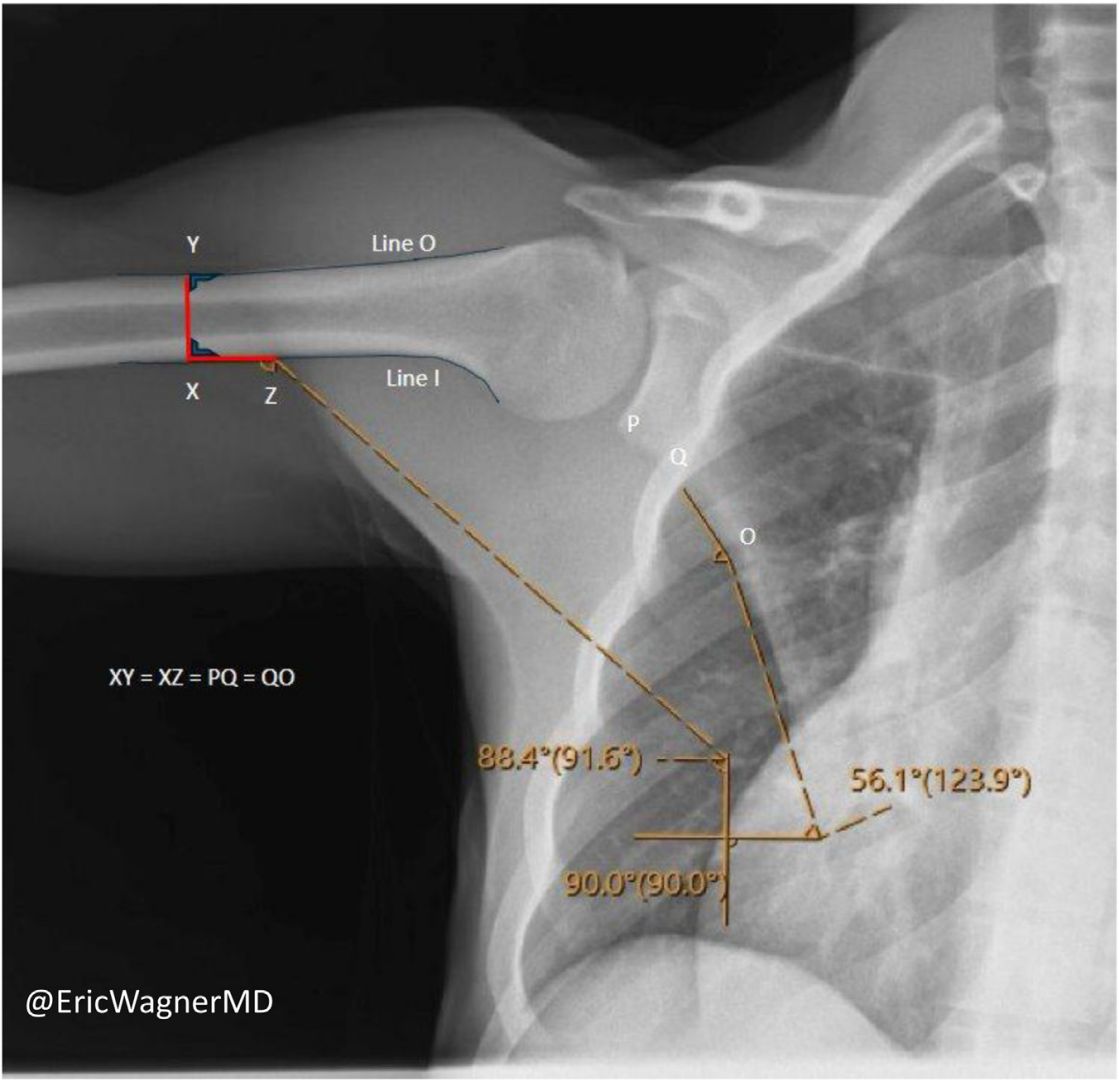
Method for measuring the SHR. *Vertical* and *horizontal* reference lines were superimposed on the image. The glenohumeral angle was determined by measuring the angle formed between the vertical reference line and a line drawn along the medial cortex of the humerus (*Line XZ*). The scapulothoracic angle was measured as the angle between the *horizontal* reference line and a line parallel to the lateral border of the scapula (*Line QO*). The lengths of *Lines XZ* and *QO* are standardized based on the diameter of the humeral shaft (*Line XY*). *SHR*, scapulohumeral rhythm.

Glenohumeral abduction, scapulothoracic rotation, and SHR were manually quantified from DDR imaging by two authors, blinded to the group of interest when measuring preoperative radiographs.^14^^,^^15^^,^^18^^,^^20^^,^^39^ PACS (Sectra Medical, Linköping, Sweden) was used to draw and measure angles on each radiograph. Vertical and horizontal reference lines were first dawn to overlay each image. The glenohumeral angle was calculated by the angle subtended by the vertical reference line and a line down the medial cortex of the humerus. The scapulothoracic angle was calculated using the angle subtended by the horizontal reference and a line parallel to the lateral border of the scapula (Fig. 3).

Angles measurements were performed in the following ranges of shoulder abduction: 0°-30°, 30°-60°, 60°-90°, and 90°-120°, to obtain an accurate representation of SHR in each interval of shoulder motion. If 0° was not visualized, the resting glenohumeral abduction was considered the starting point. Frames that are closest to the interval endpoints were used for measurements. The SHR was then calculated by dividing the change in glenohumeral abduction (ΔH) by the change in scapular upward elevation (ΔS) using the formula SHR = ΔH/ΔS across the total range of motion below 120° and between the 0°-30°, 30°-60°, 60°-90°, and 90°-120° abduction intervals.

### Subjective improvement

Patient-reported outcome measures (PROMs), including visual analog scale (VAS) pain score,^25^ American Shoulder and Elbow Surgeons (ASES) shoulder score,^36^ and the Subjective Shoulder Value (SSV),^9^ were collected for all postoperative aTSA and rTSA shoulders at a minimum of 1 year postoperative, either during clinical follow-up visits or via phone interviews for outcomes questioning.

### Statistical analysis

Descriptive statistics were used to summarize data, including percentages and counts for categorical and ordinal data and medians with interquartile ranges for continuous data. Differences between groups were analyzed using unpaired Student's *t*-tests for continuous variables, and a paired *t*-test for subgroup analyses, as well as a Bonferroni correction for multiple statistical tests. Interclass correlation of measurements was used to calculate the inter-rater reliability between the two measurers. A power analysis based on a previous study suggested that 26 patients in the aTSA group and 26 patients in the rTSA group would provide 80% power to detect a significant difference in SHR between the two groups using Student's 2-tailed t-test with a significance level of *P* < .05.^37^ All analyses were carried out using R v. 3.6.1 (R Foundation for Statistical Computing, Vienna, Austria). A *P* value of less than .05 was considered statistically significant.

## Results

### Patient demographics and operative details

Overall, 28 postoperative aTSAs, 43 postoperative rTSAs, and 32 normal controls were examined in this study. The 28 postoperative aTSA DDRs were taken from 27 patients, one of whom was bilateral. Four (14%) aTSA shoulder used a 15° half–glenoid augment, 9 (32%) used a 15° full–glenoid augment, and 15 (54%) did not use an augment. The 43 postoperative rTSA DDRs were taken from 42 patients, one of whom was bilateral. Of the 43 postoperative rTSA shoulders, 28 (65%) used an inlay design, 6 (14%) used an onlay design, and 9 (21%) used a hybrid design. Twenty five (58%) of the rTSA shoulders used a 15° full–glenoid augment, 11 (26%) did not use an augment, and 7 (16%) used humeral head–structural wedge-shaped graft with a long post.^16^ Fourteen aTSA and 14 rTSA shoulders also possessed corresponding preoperative DDRs. The 32 normal controls were taken from 29 patients, three of whom were bilateral. Study group demographics and other characteristics can be found in Table I. Of note, in the rTSA group, mean age and proportion of females was higher. Time to DDR study was higher in after aTSA.

**Table I:** Demographics data.

	aTSA (n = 28)	rTSA (n = 43)	*P* value
Age, mean ± SD (yr)	66 ± 7.9	71 ± 10.5	**.034**
Sex − female, n (%)	10 (35.7%)	28 (65.1%)	**.015**
Laterality − right, n (%)	9 (32.1%)	20 (46.5)	.229
Smoking status, n (%)			.527
Current	0	1 (2.3%)	
Former	14 (50%)	17 (39.5%)	
Diabetes mellitus, n (%)	8 (28.6%)	12 (27.9%)	.952
Time from surgery to DDR acquisition, mean ± SD (mo)	16.3 ± 13.3	12.3 ± 9.4	.133

*aTSA*, anatomic total shoulder arthroplasty; *rTSA*, reverse total shoulder arthroplasty; *SD*, standard deviation; *DDR*, dynamic digital radiography.

Bold *P* value denotes statistical significance.

### Scapulohumeral rhythm

**Figure 4:**
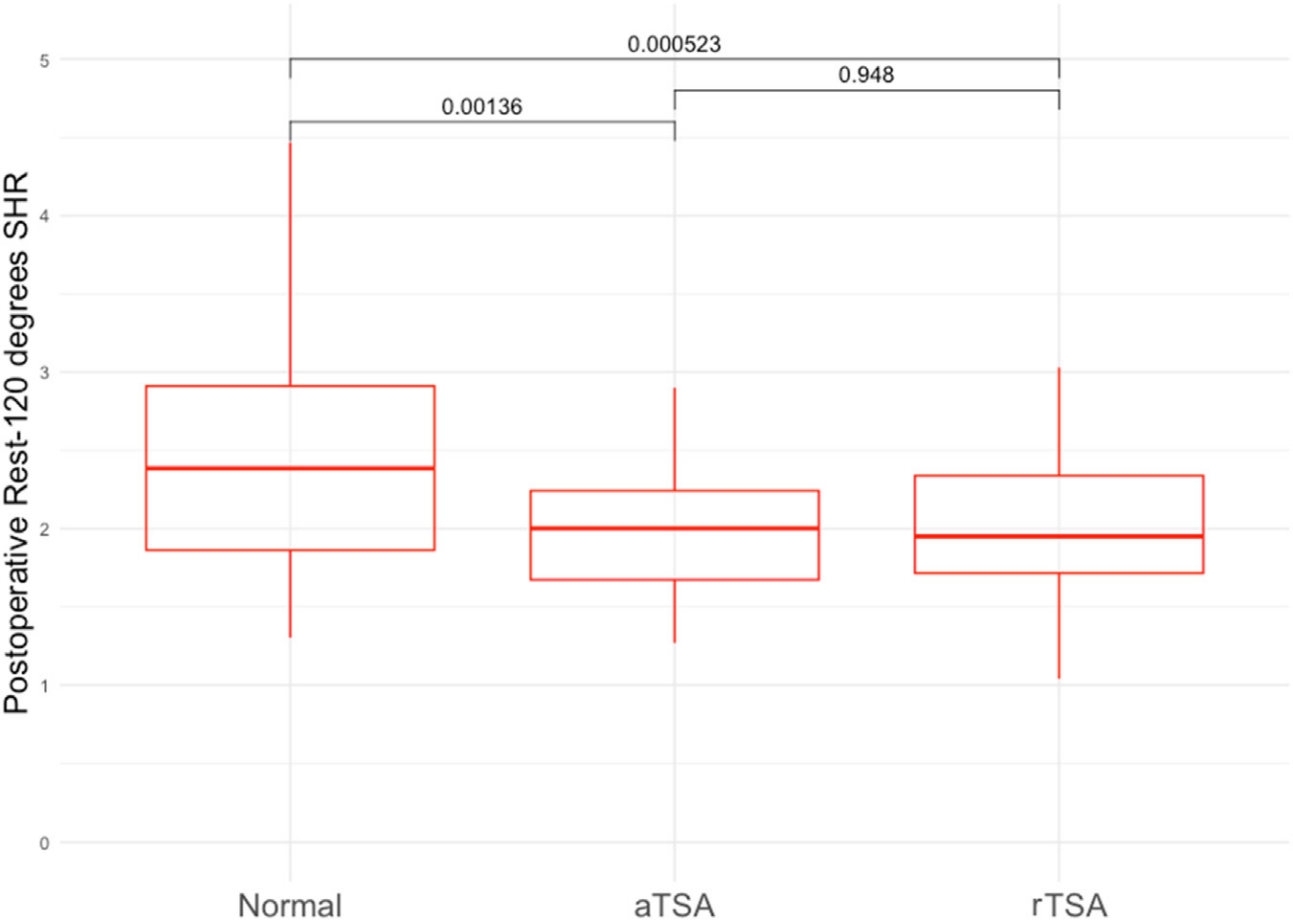
Box-and-whiskers plot comparing rest–120° range-of-motion SHR for normal controls, (n = 32), postoperative aTSA (n = 28) and postoperative rTSA (n = 43) cohorts. The normal cohort demonstrates a significantly higher total SHR compared to both operative cohorts. *Box* represents IQR (75th, 50th, and 25th percentile); whiskers represent largest value within 1.5 times IQR above the 75th or below the 25th percentile, respectively. *Dots* represent individual outliers outside of this range. *SHR*, scapulohumeral rhythm; *aTSA*, anatomic total shoulder arthroplasty; *rTSA*, reverse total shoulder arthoplasty; *IQR*, interquartile range.

Rest–120°, and 30° interval SHR between normal, aTSA, and rTSA cohorts can be found in Table II and Figure 4. There was no significant difference between the aTSA cohort's rest–120° SHR of 2.00 and the rTSA cohort's SHR of 1.95 (*P* = .948). The normal shoulder cohort possessed res–-120° SHR of 2.38, and this was higher than both aTSA (*P* = .001) and rTSA (*P* = .0005). The inter-rater reliability was excellent for glenohumeral and scapulothoracic angle measurements used to calculate the SHR (Iintraclass correlation coefficient = 0.99, 95% confidence interval 0.99-0.99).

**Table II:** SHR in normal control, aTSA, and rTSA by humerothoracic abduction interval.

Intervals of humerothoracic abduction	Normal control (n = 32)	aTSA (n = 28)	rTSA (n = 43)	*P* value
Rest − maximum (up to 120°)	2.38 (1.86-2.91)	2.00 (1.67-2.24)	1.95 (1.72-2.34)	**<.001**
Rest − 30	1.99 (1.32-3.18)	1.80 (1.43-2.64)	1.93 (1.42-2.71)	.187
30°-60°	3.34 (2.04-4.46)	1.81 (1.37-2.25)	1.93 (1.55-2.17)	**<.001**
60°-90°	3.23 (2.29-4.26)	2.21 (2.02-3.20)	1.98 (1.54-2.87)	**.004**
90°-120°	2.24 (1.20-5.89)	2.74 (1.51-3.60)	2.03 (1.69-2.60)	.550

*aTSA*, anatomic total shoulder arthroplasty; *rTSA*, reverse total shoulder arthroplasty; *SHR*, scapulohumeral rhythm.

Values reported as median (interquartile range).

Bold *P* value denotes statistical significance.

### Paired analysis

**Figure 5:**
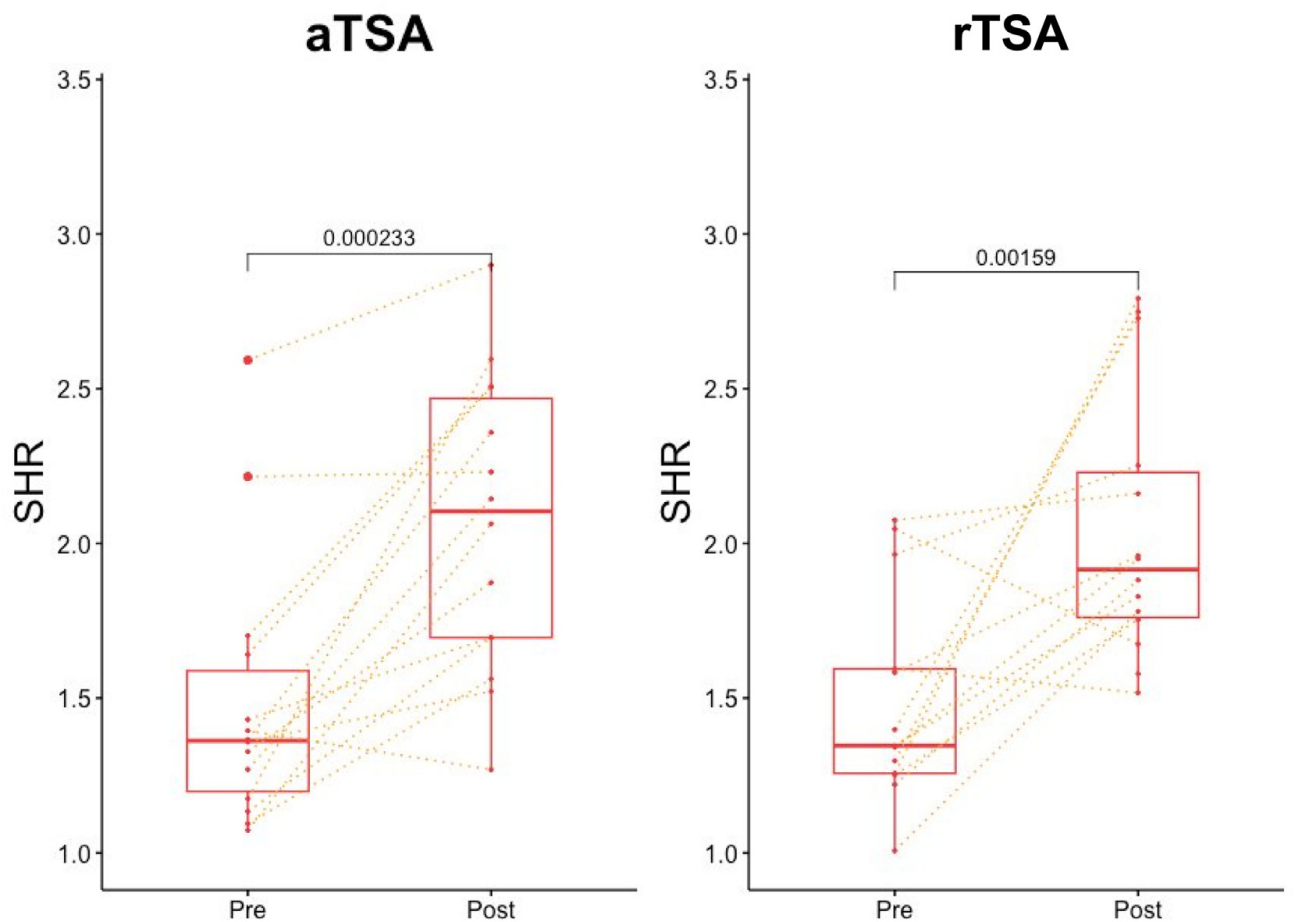
Subgroup analysis comparing changes pre- and postoperative rest–120° SHR for aTSA (n = 14) and rTSA (n = 14) cohorts. *Yellow dotted lines* track individual patients’ change in SHR following surgical intervention. *aTSA*, anatomic total shoulder arthroplasty; *rTSA*, reverse total shoulder arthroplasty; *SHR*, scapulohumeral rhythm.

A paired analysis was performed on 14 aTSA and 14 rTSA patients with both preoperative and postoperative DDRs. Preoperative rest–120° SHRs were not statistically different, with a median of 1.36 in the aTSA group and 1.34 in the rTSA group (*P* = .702). Both the aTSA and rTSA cohorts saw their median rest–120° SHR increase from 1.36 to 2.10 (*P* = .0002) and 1.34 to 1.92 (*P* = .002), respectively (Fig. 5), suggesting an increase in glenohumeral contribution to the SHR.

### Subjective improvement

Subjective PROMs were assessed using VAS pain score, ASES shoulder score, SSV, evaluated at the most recent follow-up appointment (Table III). Patients were also asked whether they would have the same procedure performed again. PROMs were obtained from all patients except one patient in the rTSA cohort, who was lost to follow-up due to being deceased. In the aTSA cohort, average final VAS pain, ASES, and SSV scores were 1.2%, 84.2%, and 93.9%, respectively, at a mean 38 months postoperative. All but one patient (96.7%) in the aTSA cohort stated they would do the same procedure again. In the rTSA cohort, average final VAS pain, ASES, and SSV scores were 1.4%, 83.9%, and 91.6%, respectively, at a mean 26 months postoperative. All but three (93.0%) patients stated they would have the same procedure again. No revisions or additional procedures were performed on any shoulders included in this study. No statistical differences were noted between both cohorts.

**Table III:** Postoperative PROMs for aTSA and rTSA cohorts.

	aTSA (n = 28)	rTSA (n = 42)∗	*P* value
VAS, mean ± SD	0.86 ± 1.56	1.40 ± 2.25	.264
SSV, mean ± SD	85.25 ± 18.63	83.90 ± 18.63	.995
ASES, mean ± SD	94.46 ± 8.57	91.57 ± 9.10	.293
Months postoperative, mean ± SD	34.89 ± 19.32	26.12 ± 12.10	**.022**

*VAS*, visual analog scale score; *SSV*, shoulder subjective value; *ASES*, American Shoulder and Elbow Surgeons; *aTSA*, anatomic total shoulder arthroplasty; *rTSA*, reverse total shoulder arthroplasty; *SD*, standard deviation; *PROMs*, patient-reported outcome measures.

Bold *P* value denotes statistical significance.

∗ One patient was deceased before patient-reported outcome measures were obtained.

## Discussion

aTSA and rTSA are both well-established treatment options for cuff-intact glenohumeral osteoarthritis, and clinical decision-making is challenging, especially for younger, more active patients. Understanding *in-vivo* shoulder biomechanics between aTSA and rTSA, using SHR as a proxy for shoulder coordination, may be helpful in determining which treatment option better restores native biomechanics, which may be an important factor in shoulder function and motion.^6^^,^^30^ Although, patients after shoulder arthroplasty are known to have a reduced SHR compared to healthy shoulders,^11^^,^^22^^,^^24^^,^^30^^,^^44^ there is very little prior knowledge on changes in scapula kinematics before and after arthroplasty to help determine whether the lower SHR is related to the preoperative pathology or the surgical intervention.^37^

Our study had several key findings. Patients that underwent aTSA and rTSA had a similar SHR in the rest–120° range, 2.00, and 1.95 respectively. These values were lower than the normal control SHR of 2.38, suggesting neither of the procedures reproduced native scapulohumeral biomechanics. In additional, in a paired analysis, SHR significantly increased from preoperative values in those that underwent aTSA and rTSA, suggesting a significant increase in the contribution at the glenohumeral joint. Finally, both surgical cohorts demonstrated excellent and similar PROMs at most recent follow-up.

The first step in restoring normal biomechanics of the shoulder is to understand normative SHR in healthy asymptomatic shoulders. Our study found SHR for normal shoulders to be 2.38, aligning with various studies that have reported *in-vivo* SHR to range from 2.2 to 2.7.^3^^,^^10^^,^^28^^,^^45^ While total SHR has been reported within this range, SHR can depend on various factors such as phase of abduction,^3^^,^^28^ arm dominance,^28^ and amount of fatigue.^41^ Likewise, the various measurement techniques in the current literature make comparisons challenging. In the presence of glenohumeral osteoarthritis, a compensatory upward rotation of the scapula has been well described, to facilitate glenohumeral and scapulothoracic abduction, and this results in a decrease in SHR.^38^ Indeed, using retroflective skin markers, Spranz et al found SHR to be inversely associated with osteoarthritis severity between 30° and 90°.^38^ Fayad et al also found decreased scapular protraction, in addition to scapula compensation, for cuff-intact glenohumeral osteoarthritis compared to normal shoulders, using electromagnetic tracking devices.^5^ These findings are consistent with our patients who preoperatively had SHRs of 1.36 and 1.34 in the aTSA and rTSA cohorts, respectively, using a dynamic radiographic technique, which we believe to be potentially more precise than skin markers.

Several groups have previously reported the scapular compensation during glenohumeral and scapulothoracic abduction in patients with aTSA, leading to a decreased SHR.^4^^,^^7^^,^^37^ Indeed, using skin sensors, Roren et al examined 14 aTSA shoulders found a postoperative SHR of 2.2 from rest to 30°, 1.1 from 30° to 60°, and 0.8 from 60° to 90°, compared to 4.4, 2.4, and 2.0 respectively, for normal controls.^37^ These findings are consistent with our study, which found patients with aTSA possessing a lower SHR of 2.00 compared to 2.38 for normal controls. However, this could be a result of the pathology or the intervention.^5^^,^^37^ Friedman et al conducted a clinical trial of 9 aTSA shoulders in 1995, finding no changes in SHR from preoperative to postoperative, suggesting normal shoulder biomechanics was not restored.^7^ However, our aTSA patients with preoperative SHRs improved their SHR postoperatively from 1.36 to 2.10, suggesting that while normal SHR was not restored, patients still achieved an increase in SHR following aTSA compared to preoperative values. It is important to note that the heterogeneous measurement techniques in the literature make comparisons challenging and are likely partially responsible for the data variability. Furthermore, implant design and surgical technique has markedly changed over the last few decades, and this could also be responsible for SHR differences independent of advances in measurement technique.

rTSA has also been associated with increased scapular contribution to shoulder abduction and therefore lower SHR.^1^^,^^11^^,^^22^^,^^24^^,^^26^^,^^35^^,^^40^ While the majority of studies are limited by study size, a meta-analysis of 4 studies found 48 postoperative rTSA patients to possess an SHR roughly 1.2 “points” less than 63 normal control shoulders.^11^ Lee et al and Reina et al noted rTSA patients possessing significantly higher scapular upward rotation, retraction, and a more prominent tilt affecting their scapular kinematics.^26^^,^^35^ This is consistent with our study, which reported a lower SHR compared to normal controls—1.95 compared to 2.38, respectively.

Current literature comparing aTSA and rTSA biomechanics is limited by low study size and using electromagnetic tracking devices that fail to show the glenohumeral and scapulothoracic contributions during *in-vivo* shoulder motion.^1^^,^^37^^,^^40^ In concordance with previous studies,^1^^,^^37^^,^^40^ we also found SHR to be significantly reduced in both aTSA and rTSA, with no differences between groups. To our knowledge, our study is among the first to also show SHR significantly improved following either aTSA or rTSA for patients with cuff-intact osteoarthritis. Likewise, this study is among the first to compare aTSA and rTSA *in-vivo* biomechanics with at least 28 shoulders in each cohort. Interestingly, we found that the aTSA cohort possessed a lower SHR than the rTSA cohort in the first half of shoulder abduction but then had a higher SHR for the second half of abduction. This indicates a transition of increased scapular contribution during the initial phase of shoulder abduction with greater glenohumeral involvement as abduction continues. This is in contrast to rTSAs, which had a relatively constant SHR throughout glenohumeral and scapulothoracic abduction. Further work is necessary to explore the clinical significance of these findings and contributing factors to variations in SHR.

Clinical comparisons between aTSA and rTSA have been performed, demonstrating clinical success and comparable outcomes for treating glenohumeral osteoarthritis with an intact rotator cuff.^8^^,^^12^^,^^33^^,^^34^^,^^42^ However, long-term clinical outcomes and complication data are still deficient. An international database study comparing rTSA and aTSA found no significant differences between clinical and radiographic outcomes, but a significantly higher complication rate for aTSA shoulders.^8^ Valsamis et al found no long-term differences between rTSA and aTSA in terms of revision surgery, serious adverse events, reoperations, prolonged hospital stay, or lifetime health care costs.^42^ These findings are in keeping with this study, in which we were not able to elicit any postoperative clinical outcome differences between aTSA and rTSA.

This study has limitations. Age was significantly different between the two groups by roughly 5 years, which likely played into the clinical decision-making, with older patients being favored for rTSA. In addition at our institution, indications for selecting rTSA over aTSA include greater than 25° of retroversion and posterior subluxation of the humerus exceeding 80°. These factors may be confounding variables when comparing SHR between groups.^21^^,^^23^ In addition, a more complete preoperative paired data set would have been preferred. The imaging methodology has some inherent limitations; notably, a 2-dimensional modality has been used to make 3-dimensional angle measurements, which may be inadvertently partially off axis, despite obtaining radiographic images perpendicular to the plane of the scapula and plane of motion. In addition, out-of-plane motion is not possible to capture; however, this did not markedly impair the ability to measure SHR in the scapula plane. Therefore, although biplane imaging techniques would be preferred, our technique permits patients to perform dynamic activities more naturally, with a larger field of view and reduces radiation exposure. The compromise also is far more feasible in the clinical workflow to obtain follow-up after surgery, rather than acquiring biplane fluoroscopy studies on postsurgical patients, which allowed us to sufficiently power this investigation. Although patients were coached by radiology technicians on cadence and effort during motion, some patients exhibited minor lateral truncal motion during abduction, and this was not accounted for in the analysis. Observing and studying the entire possible arc of motion, which could include abduction greater than 120°, could be helpful to understand overhead abilities postoperatively. Lastly, variations in how SHR is measured limit the validity of direct comparisons with previous studies.

This study assumes that restoration of more normal scapulohumeral biomechanics is desirable and potentially improves pain and function and could reduce aseptic complications; however, these are assumptions, and further work is needed to validate this and investigate its clinical significance. Initial studies have looked at the correlation between SHR and PROMs,^17^ which is an important next step, though additional studies are needed to further understand the relationship between the two. Other methodologies, such as computational models, may be more useful as they become increasingly complex and can replicate real-world scenarios. Long-term clinical data comparing aTSA and rTSA, comparing survival rates and complexity of the subsequent revision needed, would all be valuable information for clinical decision-making. Ultimately, combining all this to help inform intraoperative decisions at the index procedure and integrating this information into the planning stage for implant positioning and selection would be the “holy grail” for shoulder arthroplasty, particularly for patient-specific recommendations or planning, as well as complex scenarios like revision surgery or bone loss.

## Conclusion

Patients who underwent aTSA and rTSA for rotator cuff–intact glenohumeral osteoarthritis are associated with significantly improved SHRs from their preoperative values. However, both aTSA and rTSA SHRs remained lower than normal native shoulders. There was no difference between postoperative SHRs between rTSA and aTSA. This study suggests aTSA and rTSA partially restore coordination between the glenohumeral and scapulothoracic joints. Nonetheless, further work is needed to better understand SHR variations postoperatively, its correlation with clinical outcomes and complications, and techniques to optimize it.

## Disclaimers:

Funding: The authors report no funding was used to complete the study of interest.

Conflicts of interest: Eric R. Wagner is a consultant for Stryker Corporation, Zimmer Biomet, Acumed, and Osteoremedies. He also receives institutional research support from Konica Minolta. Michael B. Gottschalk receives research support from Stryker Corporation, Konica Minolta and Arthrex. The other authors, their immediate families, and any research foundation with which they are affiliated have not received any financial payments or other benefits from any commercial entity related to the subject of this article. Each author certifies that his or her institution approved the human protocol for this investigation and that all investigations were conducted in conformity with ethics principles of research.
